# Effects of nicomethanol hydrofluoride on dental enamel and synthetic apatites: a role for anti-caries protection

**DOI:** 10.1007/s40368-017-0314-8

**Published:** 2017-11-04

**Authors:** N. Sharkov

**Affiliations:** Department of Paediatric Dental Medicine, Faculty of Dental Medicine, Medical University, Sofia, Bulgaria

**Keywords:** Fluorine, Fluorinol^®^, Dental caries, Siliglycol, Amine fluoride, Caries prevention

## Abstract

**Aim:**

To analyse the anti-caries properties of nicomethanol hydrofluoride (NH) and the benefit of its combination with siliglycol, a coating agent.

**Methods:**

Fluoride (F) uptake by dental enamel and synthetic apatite treated with NH was measured in vitro and compared to treatment with mineral fluorides. The addition of siliglycol was also tested. The effect of NH (as a mouthwash) on salivary pH was also investigated in healthy human subjects and compared to the effect of a placebo and of nicomethanol alone.

**Results:**

In vitro experiments showed a greater and faster F uptake on dental enamel or synthetic apatite treated with NH compared to sodium fluoride. F uptake was improved further by the addition of siliglycol. In healthy human subjects, pH reduction was strongly inhibited 5 min after two mouthrinses with NH. This effect was less pronounced but still statistically significant at 15 and 30 min (p < 0.05).

**Conclusions:**

NH was able to promote the fixation of F ions and strengthen the dental structure. Its combination with siliglycol further improved F uptake by the tooth and the control/inhibition of dental biofilm development.

## Introduction

Despite a marked improvement in dental caries prevention over the last few decades, 60–90% of school children and nearly 100% of adults have dental cavities (Kimura et al. 1983). In 2020, the annual cost of dental treatment, most related to dental caries, is expected to rise to €93 billion within the member states of the European Union (Patel 2012).

Dental caries bears a significant medical, social and economic cost in children, being one of the frequent reasons for absence from school (Rugg-Gunn 2013), and the commonest reason for general anaesthesia in young children when tooth extraction is required (Banoczy and Rugg-Gunn 2013). The prevalence and severity of dental caries, which vary widely within Europe, remains a problem in socio-economically deprived groups (Kimura et al. 1983; Markovic et al. 2013).

The use of fluoride (F) toothpaste is considered to be one of the main contributing factors for the decrease in dental caries prevalence in children in both developed and developing countries (Bratthall et al. 1996; Cury et al. 2004; Marinho 2009; Marinho et al. 2013; Rugg-Gunn and Banoczy 2013). Over 90% of toothpastes sold in Europe are fluoridated (Rugg-Gunn and Banoczy 2013). Fluoride acts as an anti-caries agent by counterbalancing the mineral losses caused by acid production, mainly through the precipitation of fluoridated mineral components on teeth (Byeon et al. 2016; Lata et al. 2010; Tenuta and Cury 2010).

Various F compounds have been tested in F toothpastes, including amine F, an organic F that is widely used in Europe (Rugg-Gunn and Banoczy 2013). The solubility of the F-containing compound and its adhesion to the tooth surface determines the bioavailability of F, which is critical for caries prevention (ten Cate 1999; Galuscan et al. 2003; Priyadarshini et al. 2013; Carey 2014).

Early work suggested that amine F was superior to mineral F for reducing the solubility of the enamel, improving its F content and preventing caries (Muhlemann et al. 1968; Cahen et al. 1982; Barbakow et al. 1983; Tadmor et al. 1989). Amine F has tension-active properties which increase the affinity of F to the enamel surface and provide a sustained F release, allowing prolonged, long-lasting action (Madlena 2013). In addition, amine F exerts an anti-plaque activity by inhibiting bacterial adhesion (Shani et al. 1996; Priyadarshini et al. 2013).

Nicomethanol hydrofluoride (NH), which belongs to the amine F group, has a chemical/organic structure designed to improve the absorption of F by the phosphocalcic tooth surface. The reactivity of NH with tooth enamel and with a synthetic hydroxyapatite was first described (Szilagyi 1981). NH can be combined with siliglycol, a coating agent used in some toothpaste formulations to support F action.

The aims of the study were to investigate the intrinsic properties of NH alone and the benefit of its combination with a covering agent (siliglycol) for anti-caries protection.

## Materials and methods

In vitro and in vivo investigations were conducted to study the intrinsic properties of NH, combined or not to siliglycol.

**Fig. 1:**
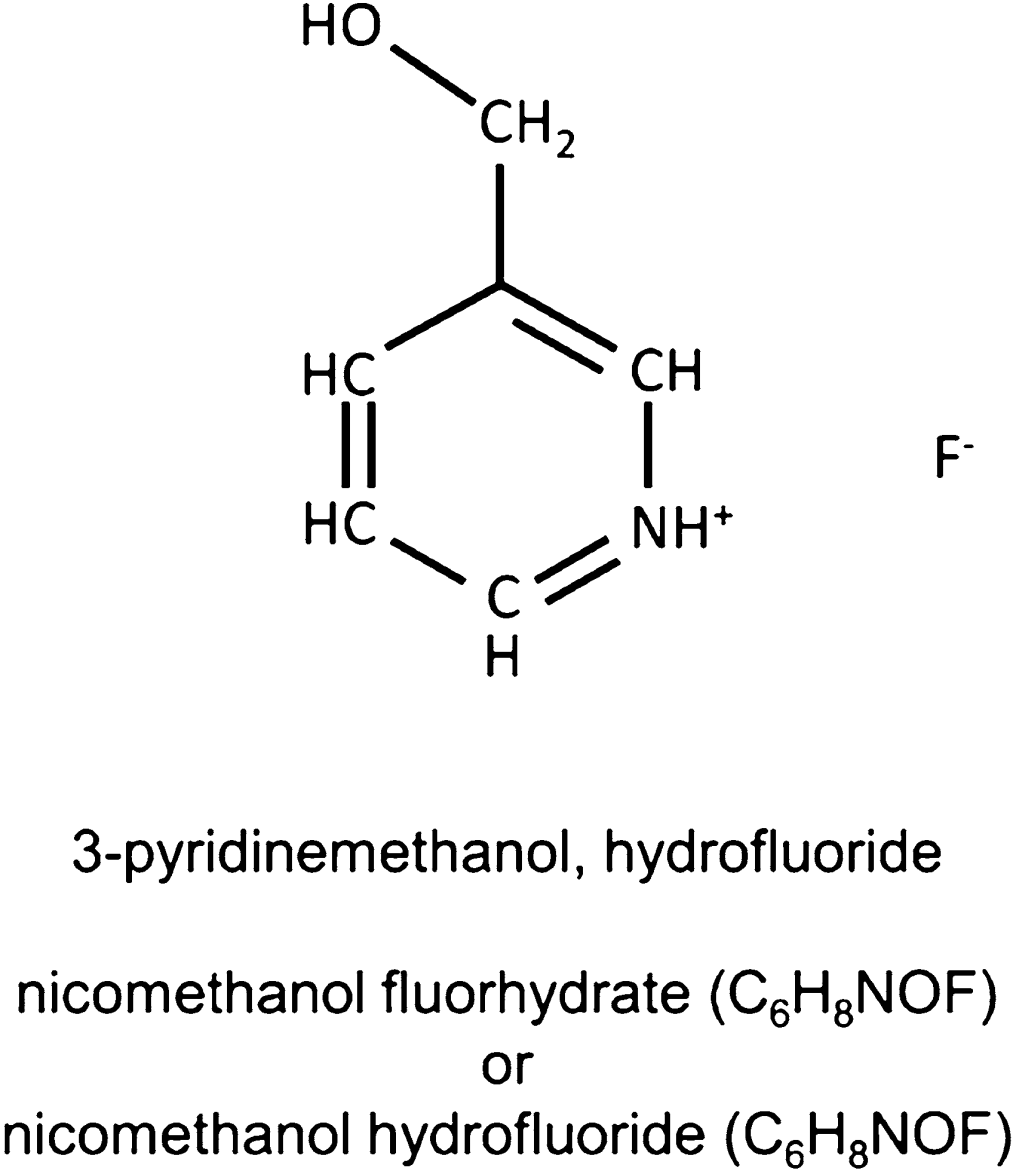
Chemical structure of nicomethanol hydrofluoride (NH)

NH (trade name Fluorinol^®^, Pierre Fabre Médicament, Castres, France) is an organic fluorine salt, belonging to the amine F group. This organic F possesses a fluoride ion (F^−^) that is ionically bound to the rest of the molecule (Fig. 1).

Siliglycol, also called PG-12 Dimethicone, is a coating agent that belongs to a group of polymeric organosilicon compounds.

### In vitro investigations

The absorption properties of NH on the surface of natural and synthetic apatites were investigated in two in vitro experiments. The addition of siliglycol to NH was considered in a third experiment.

### Calcium fluoride formation measurements were made using infrared spectroscopy

F uptake was measured at the surface of dental enamel and synthetic apatites after treatment with NH, sodium fluoride (NaF) or sodium chloride (NaCl, control solution). Two quantities of apatite were tested: 100 and 200 mg. The fluoridating solutions each contained 125 mg F^−^ per 100 mL solution. Initial pH was 5.5 and initial temperature was 20 °C. Calcium fluoride (CaF_2_) formation was measured indirectly by comparing the percentages of F ions bound by the apatites. Apatite fluoridation was measured by infrared spectroscopy according to the methodology described by Okazaki (1983). Absorption intensity was measured for the OH^−^, PO_4_^−^, CO_3_^2−^ ions and the ratios CO_3_^2−^/PO_4_^−^ and OH^−^/PO_4_^−^ were calculated.

### F uptake measurements by infrared spectroscopy and X-ray diffraction

F uptake at the surface of dental enamel was measured after treatment with either NH or NaF, both containing a proportion of 220 mg F^−^ per 100 mL solution. A test sample of 100 mg of natural dihydroxyapatite was placed under ultrasonic vibration in the presence of 10 mL of the solution to be tested and 10 mL of buffer solution at pH 5.5. After 1 min of contact, the reaction mixture was filtered to separate the fluoroapatite precipitate. The filtrate underwent the same treatment after 2 min and a new fluoroapatite precipitate was obtained. Successive filtrates after 3, 10, 15 and 30 min of contact, respectively, were isolated. The percentage of formed fluoroapatites was assessed.

Qualitative and quantitative F uptakes by enamel surfaces were measured by quantifying fluoride ions using infrared spectrophotometry (Perkin Elmer spectrophotometer, model 521) and X-ray diffraction (Guigner-de Wolff chamber, Nonius brand) (Baud and Bang 1970; Okazaki 1983).

### F uptake measurements in the presence of siliglycol

F uptakes at the surface of a synthetic carbonated apatite (structural analogue of dental enamel) were measured after treatment with NH alone or NH combined with siliglycol (Featherstone et al. 1990). The solution of NH was prepared with 4.25 mL of NH and 100 mL of distilled water. A solution of 0.5 mL of siliglycol was included to prepare the “combined” solution.

A sample of 500 mg of apatite was in contact with each of the tested solutions for 3 h. Fluoridation rate after 3 h was so high that the time of contact was reduced to 5 and 30 min. F uptake was measured at each time point.

Since the extent of F uptake was still high, the tested solutions were diluted to a factor of 10. Synthetic apatite was treated for 3 min, rinsed with water, left in contact with the solution for 1 h, in a humid environment, to allow potential fluoridation to occur and rinsed again before measuring F uptake.

Siliglycol “mechanical” retention was evaluated by a bonding test on Teflon plates. Two solutions of NH diluted by a factor of 10 were tested, with or without siliglycol. The Teflon plates were immersed in the tested solution, removed from the solution and left to dry for 1 min. The plates were then rinsed in an agitated solution, in which F uptake was measured.

### Clinical investigations

Some of the studies have been conducted in the early 1980’s and at that time, in France, ethical approval was not needed.

NH impact on salivary pH was investigated in subjects who had taken the product in mouthwash form (250 mg F^−^ per 100 mL solution). This effect was compared to the effect of a placebo and of a control (nicomethanol solution, with the same molar concentration as the tested solution). The assessment of pH change at several time points (5, 15 and 30 min) was the primary study endpoint.

A total of 12 healthy human volunteers were enrolled (no gingivitis diagnosed and systematic diseases diagnosed as well). A sample of 2–3 mL of saliva was taken at baseline. Each subject had two mouthrinses of 60 s each, separated by 1 min, using 15 mL of solution. A wash-out period (at least 3 days) separated the testing of the two different solutions. At the end of each mouthrinse, the content of the oral liquid was expectorated. Saliva samples were taken at 5, 15 and 30 min after the two 60-s mouthrinses, and pH measurements were performed on 2 mL of saliva mixed with 0.5 mL of glucose of neutral pH, every hour for 6 h (pH meter Digital Metrohm Herisau). Data were analysed using the one-way analysis of variance test.

## Results

### In vitro investigations

#### Calcium fluoride formation

With the control solution (NaCl), F uptake was 0.015% on dental enamel surfaces and 0.05% on synthetic apatite surfaces. F uptake was greater or higher with NH than with NaF solution, both on dental enamel (0.75 vs 0.06%) and on synthetic apatite (23.1 vs 1.2%). Overall, F uptake was approximately 12 times greater on dental enamel and 19 times greater on synthetic apatite, with NH compared to NaF.

**Fig. 2:**
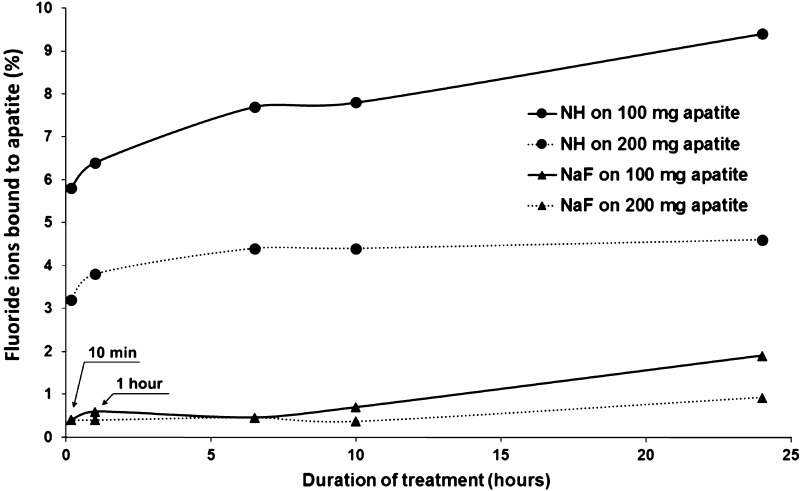
Percentage of fluoride ions (F^−^) bound by variable quantities of synthetic apatite (100 and 200 mg). *min* minute, *NaF* sodium fluoride, *NH* nicomethanol hydrofluoride

Calcium fluoride (CaF_2_) formation was greater with NH than with NaF solution, as indicated by the percentages of F ions bound by the synthetic apatite (Fig. 2).

**Fig. 3:**
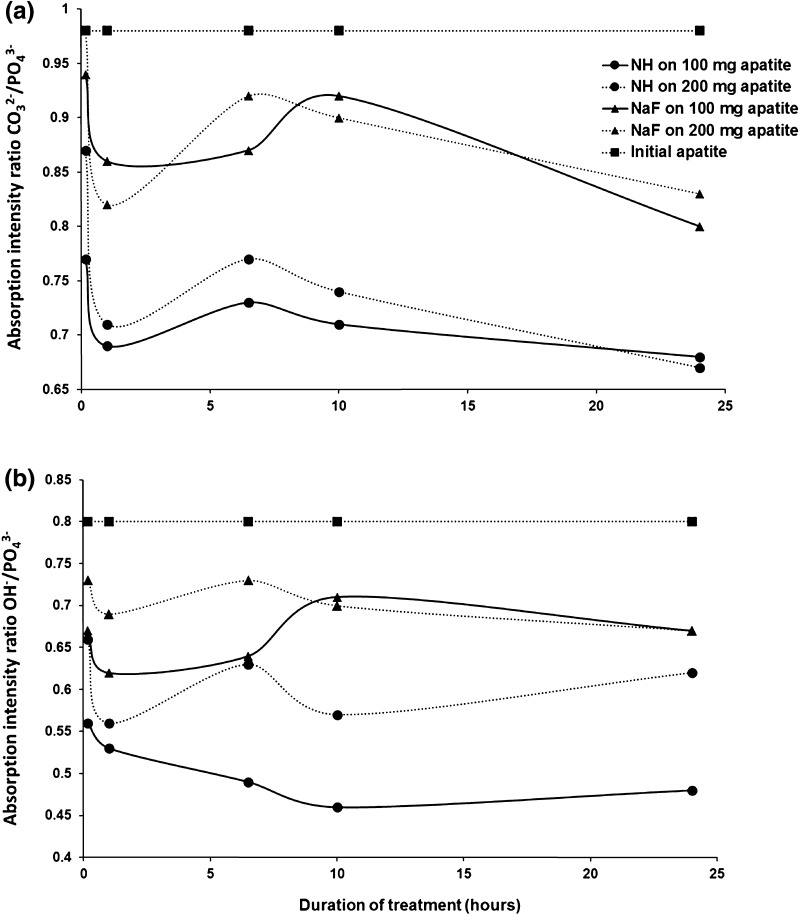
Fluoridation of synthetic apatite after treatment with solutions of nicomethanol hydrofluoride (NH) and sodium fluoride (NaF): data obtained by infrared spectroscopy. *NaF* sodium fluoride, *NH* nicomethanol hydrofluoride

Investigations using infrared spectroscopy showed a reduction of the ratios of CO_3_^2−^/PO_4_^−^ and OH^−^/PO_4_^−^, indicating a decrease in the content in OH^−^ and CO3^2−^ ions in the synthetic apatite (Fig. 3).

### F uptake measurements

**Fig. 4:**
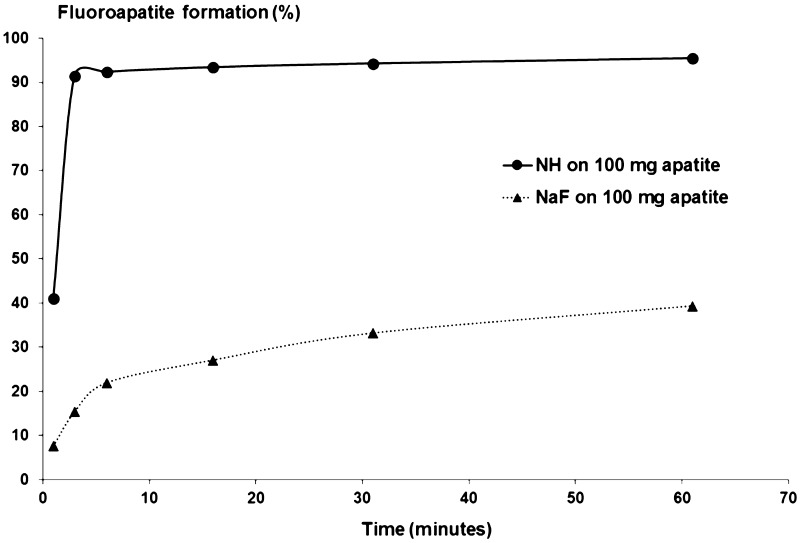
Formation of fluoroapatite after treatment with nicomethanol hydrofluoride (NH) and sodium fluoride (NaF). *NaF* sodium fluoride, *NH* nicomethanol hydrofluoride

F uptake was greater with NH than with NaF from the first minute of contact. At 1 min of contact, F uptake was 41.0% with NH vs 7.6% with NaF (Fig. 4). At 3 min of contact, F uptake was 91.5% with NH vs 15.4% with NaF. Overall, bonding of F ions to the substrate was faster and quantitatively superior with NH than with NaF (× 5.4 at 1 min of contact, and × 5.9 at 3 min of contact).

#### F uptake measurements in the presence of siliglycol

At 3 h of contact, a marked F uptake was observed (4.75–5%). This quantity is higher than the maximum theoretical quantity of F uptake (3.8%) assuming a complete fluoridation of the apatite (Ca_10_(PO_4_)_6_(OH)_2_–Ca_10_(PO_4_)_6_F_2_).

Tests on the diluted solutions showed a similar F uptake after 3 min of treatment with NH alone and with the combination of NH and siliglycol. After rinsing the apatite with water, F uptake was 13.2% higher in the sample treated with the combined solution rather than NH alone (0.302 vs 0.262, Table 1).

**Table 1:** Percentage of fluoride uptake on synthetic apatite

Solution	% Fluoride measure I	% Fluoride measure II	% Fluoride mean value
NH	0.264	0.260	0.262
NH + siliglycol	0.306	0.299	0.302

The experiment was performed twice (measure I and measure II) and the mean value was considered for analysis

*NH* nicomethanol hydrofluoride

The bonding test on Teflon plates showed a greater F uptake after immersion into the solution with siliglycol (2.74 10^−5^ mol/L) vs the fluoridated solution without siliglycol (1.87 10^−5^ mol/L). F uptake increased by approximately 30% in the presence of siliglycol.

#### Clinical investigations

**Fig. 5:**
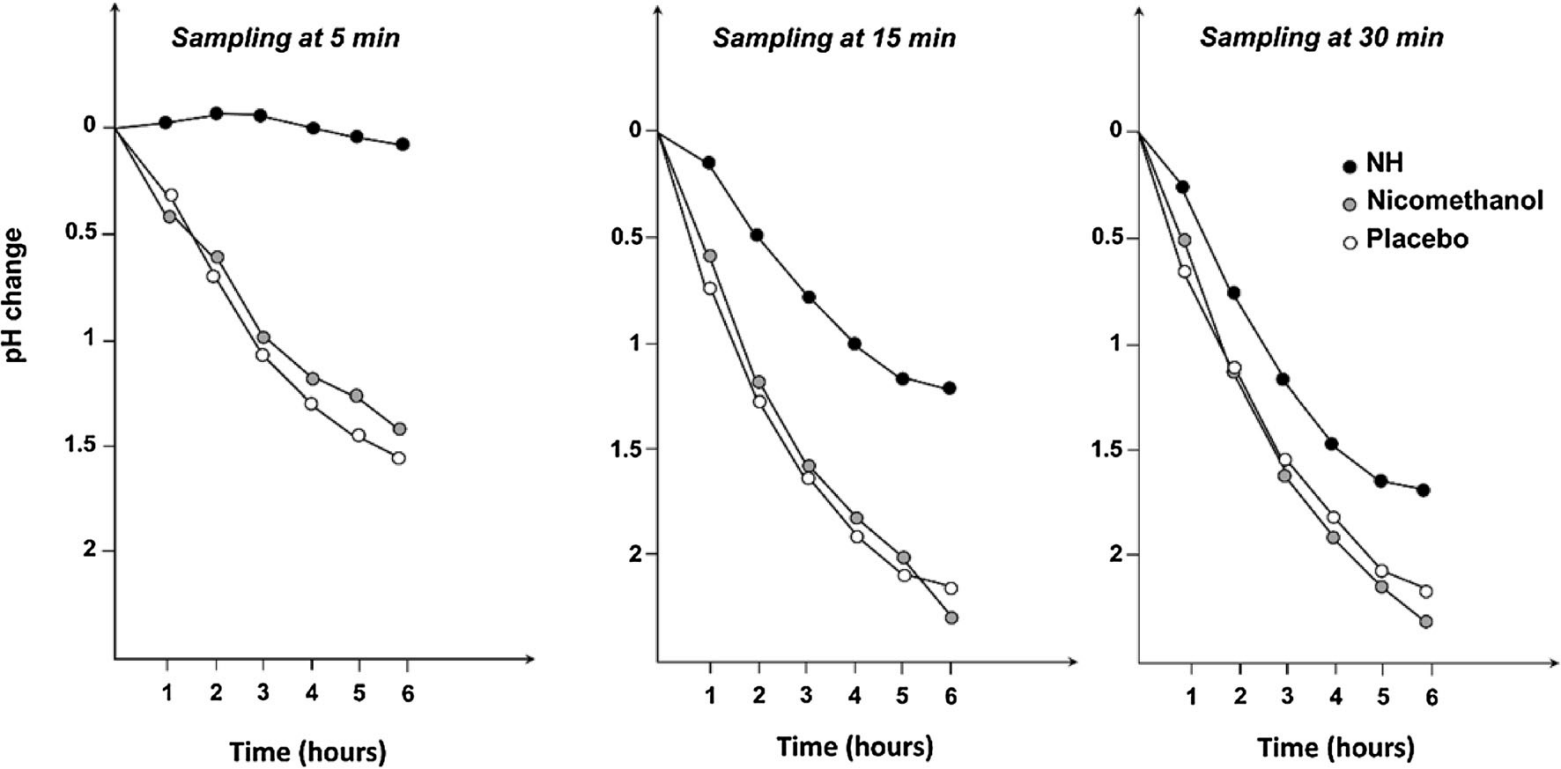
Change in salivary pH following two mouthrinses: effect of treatment and time of sampling (5, 15 and 30 min after second mouthrinse). *min* minute, *NH* nicomethanol hydrofluoride

For healthy human volunteers, the pH reduction following two mouthrinses was smaller with NH than with the control solution (nicomethanol alone) or with the placebo. A marked inhibitory effect of NH on salivary glycolysis was observed 5 min after the mouthrinse (Figs. 4, 5). After 15 and 30 min, the effect appeared to be less pronounced but remained statistically significant (p < 0.05, Fig. 5).

## Discussion

Treatment with NH alone was associated with a greater and faster F uptake on dental enamel surfaces or synthetic apatite surfaces than treatment with NaF. The addition of the coating agent siliglycol improved F uptake even further. NH was also shown to exert a strong and sustained inhibitory effect on salivary glycolysis in healthy human volunteers. All together, these findings support the anti-caries activity of NH.

Fluoridation rate has been recognised as an important aspect of the caries preventive action of F (Galuscan et al. 2003). Amine derivatives in the form of hydrofluoride are excellent carriers for F ions, which can be exchanged with hydroxyl groups from the hydroxyapatite of enamel. In vitro experiments showed that this exchange is very fast, with 50% of F ions being picked up by the apatite after 1 min of interaction.

The faster F uptake with NH than with NaF could be explained by the molecular structure of the compound. The presence of both a hydrophilic part (amine group) and hydrophobic part (hydrocarbon chain) in amine fluorides reduces surface tension of liquids and increases the affinity of F to the enamel surface. As a result, more F ions are available on the enamel surface, providing better conditions for F uptake.

F uptake is not only faster but also greater with NH than with mineral fluorides, i.e. approximately 5 times greater compared to NaF after 1 min of contact. Apatite fluoridation was clearly shown by infrared spectroscopy, which revealed a decrease of OH^−^ and CO_3_^2−^ ions in the apatite. F uptake obtained with NH was higher than expected with a total exchange of hydroxyl ions by fluoride ions, suggesting CaF_2_ formation.

The formation of CaF_2_ was confirmed by an in vitro study (Lacout 2011) that investigated the fluoridation capacity of different fluoridated solutions compared to a control solution of NaF (250 ppm of F^−^) at a pH 4.33. Synthetic hydroxyapatite discs (similar to dental enamel) were treated with 6 mouthrinses and a control solution, with a contact duration of 2, 4 and 6 h corresponding to a cumulative 2-min daily rinsing of mouthrinse used for 2, 4 and 6 months. Fluoridation was more effective with the solutions containing an organic fluoride (NH) compared to the control NaF-based solution, and this superiority increased with contact duration. Calcium fluoride structure was confirmed by X-ray diffraction.

The extent of fluoridation obtained with organic F was therefore caused by a combination of hydroxyapatite fluoridation and formation of CaF_2_ (Trombe et al. 1982; Galuscan et al. 2003).

It was suggested that the formation of CaF_2_ may be involved in mechanisms of surface remineralisation within dentine, playing a desensitising role by blocking dentinal tubules (Trombe et al. 1982), but also a protective role by decreasing the solubility of enamel in an acidic environment. In support of this hypothesis, in vitro studies on human teeth showed that NH decreased the enamel solubility to a greater extent than mineral fluorides (Vezin et al. 1985; Tadmor et al. 1989). As an illustration, the quantity of calcium and phosphorus collected after acid attack simulation (hydrochloric acid for 4 h) on healthy teeth was lower after treatment with NH rather than NaF (Vezin et al. 1985).

The decreased solubility of enamel was primarily supported by the formation of fluoroapatite and CaF_2_, which are more resistant to acid attack than hydroxyapatite.

Calcium fluoride, as a protective, adhesive layer on tooth enamel, represents a long-lasting source of F ions. Acid attacks cause these ions to be released, which promotes remineralisation (Galuscan et al. 2003). The formation of fluoroapatite and CaF_2_ enables dental enamel to resist acid attacks for a longer period of time. A pH cycling study confirmed the effects of NH on de- and remineralisation phases of advanced (> 150 µm) enamel lesions (ten Cate et al. 2008). NH was compared to NaF at variable concentrations of fluoride (0–5000 ppm F), and to a fluoride-free control solution. Treatments with 5000 ppm F both significantly enhanced remineralisation and inhibited demineralisation when compared to treatments with 1500 ppm F. The calcium apposition and loss were similar between the two sources of fluoride, except slight differences in favour of NH for demineralisation at 500 ppm and of remineralisation at 5000 ppm. At equal concentrations, the calcium loss/gain ratios were more favourable with NH than with NaF, reflecting a better balance between the de- and remineralisation phases of enamel lesions with NH.

The study conducted in 12 healthy human subjects showed that mouthrinses with NH resulted in a stronger inhibition of salivary glycolysis than the placebo or control solution (nicomethanol alone). The inhibitory effect was efficient within the first 5 min and remained significant for 30 min after the rinses. This anti-glycolytic activity strengthened the anti-caries role of NH.

The coating agent siliglycol was able to prevent the immediate elimination of fluoride following rinsing with water. This effect was confirmed by the bonding test performed on Teflon plates, which showed that the coating agent ensured a greater retention (30%) of NH. The synergetic effect of the NH/siliglycol combination was illustrated by the significant increase (13%) in F uptake after rinsing the apatite with water, in comparison to NH alone.

Siliglycol was shown to prevent bacterial adhesion on the surface of synthetic apatite (Dorignac 1996), on dental enamel (Roques et al. 2001) and on a model of the oral cavity (Zampatti et al. 1994). These findings are relevant considering that dental plaque control plays a key role in oral hygiene, especially in the prevention of caries (Roques et al. 2001), and that the first step of the plaque formation is the adhesion of bacteria belonging to the *Streptococcus mutans* group to the tooth surfaces (Furiga et al. 2008).

Using calibrated discs of dental composite (Heliomolar^®^), which has been shown to promote microorganism adhesion, the quantity of bacteria was shown to increase as the quantity of siliglycol covering the disks decreased (Dorignac 1996). Siliglycol seemed to be an effective interface for preventing bacterial adhesion to its support surface and remained sufficiently present on the support material surface to ensure that the material was protected from acid attack (Dorignac 1996).

Similar results were obtained with a toothpaste combining NH and siliglycol for the prevention of *S. mutans* adhesion to the enamel surfaces (Roques et al. 2001). The toothpaste treatment combining NH and siliglycol led to a larger decrease in the number of adherent bacteria in comparison to NH placebo, siliglycol placebo and total placebo (p ≤ 0.01). The duration of treatment (30 s vs 5 min) did not affect the results, indicating a rapid effect of the toothpaste with little or no time dependency. These results were confirmed using an in vitro artificial mouth model (Roques et al. 2001).

Similar experiments were conducted using an artificial model of the oral cavity, which was based on the continuous irrigation of bovine tooth samples with artificial saliva (Zampatti et al. 1994). Treatment with a toothpaste containing NH and siliglycol was shown to inhibit bacterial colonisation on enamel surfaces. Overall, siliglycol was found to be an effective coating agent that acted with NH to condition the surface of the tooth. The results illustrated the two main roles of the toothpaste: the elimination of plaque during brushing and the prevention of the formation of new plaque, both on permanent and primary teeth.

The synthesis of insoluble glucan from glucosyltransferases of *Streptococcus* spp. significantly contribute to bacterial colonisation, making this enzymatic activity a potential target to control the formation and development of oral biofilms. The ability of NH (10.2 mg/mL) to impair multispecies biofilm formation (Guggenheim model) was investigated by measuring the quantity of sugars released and the quantity of insoluble glucans synthesised (Furiga et al. 2014). Results are presented as percentage of enzymatic activity with respect to controls (without any compound). NH showed a significant inhibitory activity of 20.6% against the quantity of sugars released (p < 0.01), but no effect on insoluble glucan synthesis was observed (Furiga et al. 2014).

## Conclusions

NH has intrinsic properties capable of promoting anti-caries protection through various mechanisms. The present in vitro experiments showed a greater and faster F uptake to dental enamel or synthetic apatite treated with NH compared to sodium fluoride, thus suggesting an important role for NH during remineralisation phases in enabling better resistance to aggressions (such as acid dissolution) and promoting the fixation of F ions within the dental enamel. These properties have been observed from concentrations of 250 ppm of F ions and provide a protective effect on global dental structures (enamel and dentine). Moreover, NH was shown to exert a strong and sustained inhibitory effect on salivary glycolysis in healthy human volunteers, further supporting the conclusion that NH has an anti-caries function. The combination of NH with the covering agent siliglycol has also been evaluated. In vitro experiments using synthetic apatite as an analogue of dental enamel showed a marked increase in F uptake on apatite surfaces in the samples treated with a combination of NH and siliglycol as compared to NH alone. This agent therefore provides higher resistance to acid attacks and the control/inhibition of dental biofilm development.
